# Vaccination process of immunocompromised patients in the Netherlands: Current challenges and potential solutions

**DOI:** 10.1016/j.jvacx.2023.100340

**Published:** 2023-06-27

**Authors:** Cindy R. de Laat, Joost J.M. Simons, Tjalke A. Westra, Mickaël Hiligsmann

**Affiliations:** a Faculty of Health, Medicine and Life Sciences, Maastricht University, Maastricht, The Netherlands; b Market Access Department, GSK, Van Asch van Wijckstraat 55h, 3811 Amersfoort, The Netherlands; c Department of Health Services Research, CAPHRI, Care and Public Health Research Institute, Maastricht University, Maastricht, The Netherlands

**Keywords:** Immunocompromised patients, Infectious diseases, Vaccination, The Netherlands, Challenges, Qualitative research

## Abstract

**Introduction:**

Immunocompromised patients (ICP) have an increased risk for infectious diseases. Vaccines could help reduce this risk. However, ICP do not always receive or are reimbursed for the specific vaccinations on which they are dependent in the Netherlands. This research aims to gain insights into the current challenges in the vaccination process of ICP in the Netherlands. Moreover, it aims to explore potential solutions for these challenges and the ideal vaccination process.

**Methods:**

Twelve semi-structured interviews with relevant stakeholders were conducted. Partly based on the challenges found by the Council for Health and Society (RVS) and the Dutch National Health Care Institute (ZIN) an interview guide was developed.

**Results:**

Several newly emerged challenges were identified: fair reimbursement for the efforts of stakeholders; circular reasoning of vaccine reimbursement and guidelines; suboptimal translation from guidelines to practice and no smooth-running infrastructure. Most challenges corresponded with those stated by the RVS and ZIN. Affordability and knowledge deficit in healthcare providers and patients were the most important challenges. Rarely the same solutions were mentioned, and no ideal vaccination process emerged.

**Conclusions:**

The various challenges in the vaccination process of ICP in the Netherlands implies the difficulty to solve the problem. It is recommended to focus on solving the most important challenges. A potential solution is to adjust the GVS registration process to make it easier for the pharmaceutical industry to apply for reimbursement towards various high-risk groups. Additionally, vaccination should have a more prominent role in the education of healthcare providers. Furthermore, stakeholders need to cooperate more to solve the reimbursement and guidelines issue.

## Highlights

• Various challenges in the vaccination process of Dutch immunocompromised patients.
• Affordability and knowledge deficit were the most important challenges.
• Stakeholders want a fair reimbursement for their efforts to improve the process.
• Stakeholders need to cooperate more to solve the reimbursement and guidelines issue.
• Rarely the same solutions to challenges in ICP vaccination process were mentioned.

## Introduction

During the last decades, a rapid development of immunosuppressive therapies has led to a large increase in the prevalence of immunocompromised patients (ICP) [1], [2]. An underlying disease or a treatment with immunosuppressive therapies can cause a compromised state of the immune system. Therefore, ICP have heterogenous clinical conditions like primary immune deficiency, a removed spleen, human immunodeficiency virus (HIV) or cancer [2], [3], [4], [5], [6], [7]. ICP have an increased risk for infectious diseases compared to immunocompetent people, including vaccine preventable infections, demonstrating both an increased attack rate and an enhanced risk to develop complications or severe diseases [3], [8]. Vaccination is an often-mentioned strategy in guidelines to prevent infections in ICP [9], [10]. However vaccination recommendations for ICP differ per condition, e.g. considering spleen removal vaccinations against S. pneumoniae, H. influenzae type b and Neisseria meningitidis should be provided [11], [12]. Vaccinations should be provided with caution, since an immune deficiency could result in reduced or limited response to vaccinations and timing of vaccination is important [8], [13], [14], [15]. Maintaining herd protection by means of high public vaccination coverage is necessary to protect ICP [4]. So, ICP are a medical high-risk group for infectious diseases and are therefore dependent on specific vaccinations [3], [5], [16]. If ICP do not receive these vaccinations, they have a serious increased morbidity and mortality risk after infectious disease related complications [1], [5].

Despite this, Dutch research showed that only 30% of the patients after spleen removal received vaccination according to the guidelines of the Dutch National Institute for Public Health and the Environment (RIVM) [12]. Moreover, the Dutch Council for Health and Society (RVS) stated that there is hardly any vaccination in regular healthcare, which especially applies for ICP [17]. The Dutch National Health Care Institute (ZIN) supports this by indicating that ICP do not always receive or are not always reimbursed for specific vaccinations on which they are dependent [11]. So, improving the vaccination process of ICP to prevent health loss is highly needed, since approximately 0.2% (approximately 280,000 persons) of the Dutch population were ICP in 2016 and this number is increasing [1], [2], [4], [18]. The vaccination process of ICP could be improved by obtaining better insights into the current challenges and their potential solutions.

To date, there is limited research conducted in that field. Dutch research by Doornekamp et al. (2019) found that vaccinations were not discussed by default during consultations between hospital healthcare professionals (HCPs) and ICP. Furthermore, HCPs indicated that they experienced barriers concerning vaccinating their ICP, like timing issues, logistical obstacles, and financial problems [19]. Doornekamp et al. (2019) proposed to actively track down barriers and solve them in a multidisciplinary approach. The RVS (2021) indicated several challenges regarding vaccination in regular healthcare, such as a focus on curative care instead of preventive care. Furthermore, the RVS determined a knowledge deficit in healthcare providers and patients, because little attention was paid to good vaccination care in the education of HCPs. Medical guidelines were often insufficiently up-to-date and information to patients was frequently insufficient and inactive. Lastly, there was no optimal registration of risk factors in healthcare and Information and Communications Technology (ICT)-systems of the various healthcare providers could not communicate well with each other. This made it difficult to target high-risk groups for vaccination [17]. The ZIN concluded in an exploratory analysis that in four aspects (recognizability, affordability, feasibility and awareness) of healthcare there were bottlenecks for vaccinating ICP [11]. Recognizability entailed that vaccination guidelines were unclear. The next identified aspect was affordability. The ZIN concluded that some effective vaccines were not reimbursed from the basic insurance package. Moreover, some pharmaceutical companies did not (properly) apply for reimbursement of their vaccine for ICP. Additionally, assessing vaccines based on evidence is perceived as difficult, due to a relatively small amount of each patient group. Next feasibility, which indicated that there was ambiguity about which healthcare provider was responsible for providing and administering the vaccines. Furthermore, vaccination could not be possible due to a lack of vaccines availability, the window of opportunity to vaccinate ICP expired, or patient’s vaccination refusal. Moreover, there was unclarity about the reimbursement. The last aspect concerned awareness, which entailed that patients and healthcare providers were not aware of the medical necessity of the vaccinations resulting in possible knowledge deficit [11]. There are some similarities in the RVS and ZIN challenges as the RVS have related their challenges as much as possible to the challenges mentioned by the ZIN [17].

New research could verify the research findings of the RVS and ZIN, thereby increasing the credibility of their findings. The primary aim of this study was therefore to gain insights into the current challenges in the vaccination process of ICP in the Netherlands. Furthermore, this study also aimed to explore potential solutions for the challenges and the ideal vaccination process.

In the Netherlands, several approaches for the provision of vaccinations can be distinguished. The Netherlands has a National Immunization Program based on collective prevention and reimbursement by the state [20], [21]. Persons with an increased risk of severe form of flu, like ICP, can receive a flu vaccination free of charge at the GP practice [21], [22], [23]. Moreover, people can voluntarily purchase approved vaccinations prescribed by a GP or vaccination center, like the Municipal Public Health Service (GGD) [21], [24], [25]. Some vaccinations for ICP are part of a hospital treatment [21]. This is largely reimbursed by the mandatory basic health insurance package, via a case-based system called diagnosis-treatment combination (DBC), after the annual deductible (€385) is paid [26], [27]. The expensive medicines, if costs per patient per year are >1000€, are funded by add-ons [27]. ICP can also receive vaccinations that are not part of hospital treatments. These vaccinations qualify only for reimbursement from the basic health insurance package when they are part of the medicine reimbursement system (GVS) [28]. Most vaccines included in the GVS are only eligible for reimbursement if they are given to certain high-risk groups [17]. After approval for market authorization for a specific target group, the pharmaceutical company can compile a report which could be used by the ZIN to assess whether the vaccine can be reimbursed from the basic health insurance package. Based on the advice of the ZIN the Minister of Medical Care makes the final decision on whether or not to reimburse the vaccine [29].

**Fig. 1:**
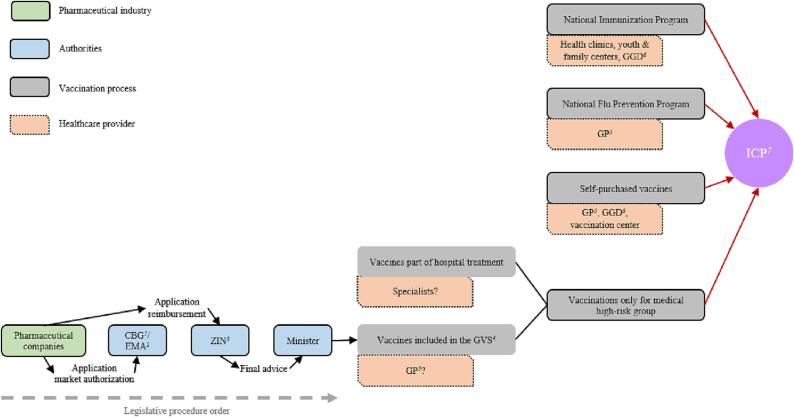
*Summary of ICP vaccination processes in the Netherlands.*^1^CBG, Dutch Medicines Evaluation Board; ^2^EMA, European Medicine Agency; ^3^ZIN, Dutch National Health Care Institute; ^4^GVS, Medicine reimbursement system; ^5^GP, General practitioner; ^6^GGD, Municipal Public Health Service; ^7^ICP, Immunocompromised patients.

So, the way the vaccinations are reimbursed depends on who administers the vaccines and the location where vaccines are administered [17]. The GP could declare the act of administering but the patient must purchase the vaccine and reimbursement of this depends on the GVS [28], [30]. Vaccines given in inpatient care are reimbursed from the hospital budget [31]. In the Netherlands, only physicians are allowed to prescribe vaccinations and by law is defined who may administer vaccines [32], [33]. Fig. 1 shows a summary of ICP’ vaccination processes in the Netherlands.

## Methods

This research was of exploratory nature and followed a qualitative design. The research was approved by the research ethics committee of Maastricht University (FHML/HPIM/2021.041).

### Data collection

Individual in-depth, semi-structured interviews with relevant stakeholders were conducted to gain insights into the current challenges and their potential solutions in the vaccination process of ICP in the Netherlands. Based on generated knowledge, an interview guide was created to enhance that the same set of topics were discussed with every participant (see Appendix A) [34]. Three other researchers reviewed and validated the interview guide. The pre-defined topic list targeted stakeholders’ insights into the current challenges and their potential solutions in the vaccination process of ICP. This was partly based on the challenges indicated by the RVS and ZIN. Only the exploratory analysis of the ZIN was used for the topic list, as the advice report of the ZIN was published after the interviews were conducted. Considering time, only the most important challenges indicated by the RVS and the ZIN were discussed with the stakeholders. Furthermore, the topic list aimed to identify the stakeholders’ awareness of the problem; what is going well in the vaccination process; the stakeholder’s ideal vaccination process; what the stakeholder and the pharmaceutical industry could to do improve the vaccination process.

The semi-structured interviews were conducted in Dutch because the interviewees and interviewer were native Dutch speakers. Each interview was recorded upon given written informed consent. The participants spoke on behalf of themselves and not on behalf of the organization or professional association they work for. The interviews were conducted from the 19th of April 2021 until the 18th of May 2021. All interviews were conducted online via Microsoft Teams with an average duration of 38 min.

Based on previous qualitative research this research targeted to conduct about 10–15 interviews. After this set data saturation was checked. The sampling goal was to recruit stakeholders in the vaccination process of ICP. This study would have sufficient participants when every stakeholder group is represented. By using purposive sampling, the first interviewed stakeholders were derived from Fig. 1 (healthcare providers, pharmaceutical industry, authorities and GGD). Additional theoretical sampling was applied and based on emerging findings a new stakeholder (pharmacist) was identified and recruited [34]. One employee working for an advisory body for the authorities was also interviewed. To ensure the anonymity of the stakeholders, the description of stakeholder roles is not provided in detail. However, in each type of stakeholder category different kinds of stakeholders were interviewed to cover multiple aspects. ICP were not interviewed due to legislative barriers [35].

### Data analysis

After transcribing the interviews verbatim, the recordings were deleted [36]. Data was handled with confidentiality as the participants remained anonymous in the data analysis. Summaries of the transcripts were sent to the stakeholders for the member check, which increases the study’s credibility [37]. The NVivo 12 software was used to analyze the data. First a deductive approach was applied, and a predefined codebook based on the interview guide was created (see Appendix B). Consequently, an iterative inductive approach consisting of three stages was applied. The first stage was open coding, which entailed line by line coding whereby concepts were identified and put into subcategories. The second stage was axial coding in which relationships were identified between the categories. Lastly selective coding involved identification of the core messages from the data and this was related to other categories. Afterwards, a theory was identified [38]. More details of the final topics can be found in Appendix C. Coding was considered finalized when no new concepts emerged from reviewing the collected data [39]. Outcomes of the interview were systematically compared with one another within each code. The first author did not have much experience with the topic before starting this research. Therefore, she was able to analyze the data with a fresh perspective. The other authors had experience with the topic and provided guidance through the study.

Moreover, quotes from the interviewees were used to illustrate that the findings emerged from the collected data rather than from the researcher’s own predispositions [37].

## Results

### Stakeholders characteristics and awareness

In total, 35 stakeholders were approached via email of which 12 participated in the interviews. The interviewees were four healthcare providers (medical specialists and GP), one pharmacist, two pharmaceutical industry employees, three governmental employees, one person working at an advisory body for the authorities and one GGD employee. 7 interviewees were female, and 5 interviewees were male. One healthcare provider and the pharmacist admitted to not being completely aware of the problem that the ICP do not always receive or are not always reimbursed for the specific vaccinations on which they are dependent. The other stakeholders were fully aware of the problem.

### Aspects that are going well

More awareness for vaccinations emerged as an aspect that is going well in the vaccination process of ICP as eight of the twelve stakeholders indicated this. According to five stakeholders, some vaccines are reimbursed nowadays. Furthermore, the national vaccinations programs are frequently mentioned as positive. One healthcare provider mentioned *“I think the National Immunization Program is followed reasonably well. So, a reasonable percentage of the people who come to us have already had those vaccinations.”*

### Challenges

Table 1 provides an overview of all the challenges in the vaccination process of ICP in the Netherlands. Seven new challenges that were not indicated by the RVS nor the ZIN emerged. For instance, stakeholders want a fair reimbursement for their efforts to improve the vaccination process of ICP. One healthcare provider indicated that drawing up guidelines falls under unpaid overtime for healthcare providers.

**Table 1:** Challenges in the vaccination process of ICP.

	Challenges (Number of stakeholders who indicated this challenge)
Newly emerged challenges	Stakeholders want a fair reimbursement for their efforts (4)
	Circular reasoning of reimbursement of vaccines and medical guidelines (4)
	Translation from guidelines to practice (3)
	No smooth-running infrastructure (2)
	Lack of hospital nurses (1)
	Pharmacists have a cumbersome way of declaring the vaccines (1)
	Lack of international collaboration (1)
Challenges indicated by the RVS^1^	Knowledge deficit in healthcare providers and patients (6)
	There is no optimal registration of risk factors in healthcare and ICT^2^-systems of various healthcare providers cannot communicate well with each other (6)
	Guidelines for GPs^3^ and medical specialists are often insufficiently up-to-date (5)
	Currently, the focus is mainly on curative care instead of preventive care within regular healthcare (3)
	Information to patients is frequently insufficient and inactive (1)
Challenges indicated by the ZIN^4^	Feasibility (11)
	Affordability (9)
	Awareness (7)
	Recognizability (5)

1 *RVS* Council for Health and Society.

2 *ICT* information and communications technology.

3 *GP* general practitioner.

4 *ZIN* Dutch National Health Care Institute.

Furthermore, four stakeholders indicated a challenge regarding the reimbursement of vaccines and the medical guidelines. One healthcare provider stated that it is hard to include a non-reimbursed vaccine in a medical guideline. Physicians will follow the guidelines. Consequently, physicians must advise vaccines that will not be reimbursed for patients. A physician could feel he is advising some quackery because the vaccines are not reimbursed. Furthermore, it is unpleasant for a physician to advise non-reimbursed vaccines to a patient when the physician suspects the patient could not afford these. However, one healthcare provider thought that about 80 to 90 percent of the ICP gets informed about the vaccines.

*“I think the reimbursements also works psychologically for the guideline makers. Officially not, of course, because they must use the literature. But if it is reimbursed and everyone is going to prescribe it and use it, it will reach the guidelines faster”*. (GGD)

**Fig. 2:**
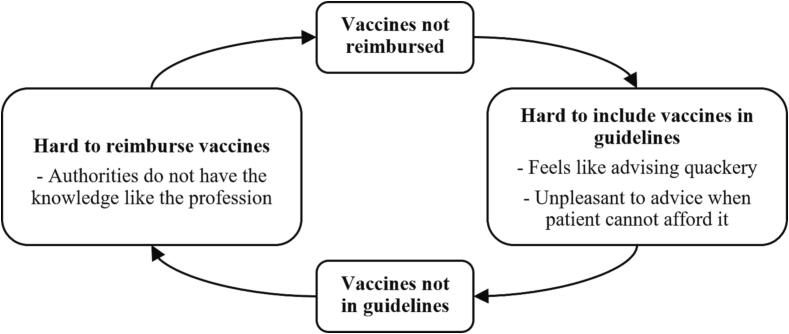
Circular reasoning of the vaccine reimbursement and the medical guidelines.

Contrarily, one authority employee mentioned that if the non-reimbursed vaccines are included in the guidelines, these would be more likely reimbursed: *“we assess the vaccines, but we will never have the knowledge like the profession itself has.”* So, you keep reasoning in a circle, which is illustrated in Fig. 2.

Another newly emerged challenge is suboptimal translation from guidelines to practice, which was indicated by three stakeholders. A pharmaceutical industry employee and authority employee mentioned infrastructure as a new challenge as the vaccination process of ICP does not run smoothly. Newly emerged challenges mentioned by one stakeholder included hospital nurses’ shortage, pharmacists having a cumbersome way of declaring the vaccines and a lack of international collaboration.

However, of all challenges mentioned by the stakeholders, most challenges corresponded with the challenges found by the RVS and the ZIN. Out of the challenges indicated by the RVS, stakeholders mentioned the ICT-issues and the knowledge deficit in healthcare providers and patients the most. Regarding the ICT-issues, mostly the communication between healthcare providers and patient data registration, like registration of the vaccinations for adults, were mentioned. Feasibility, affordability, and awareness, indicated by the ZIN, were mentioned by the majority of the stakeholders. Regarding feasibility, three stakeholders indicated a challenge concerning the implementation of the reimbursement system. ICP could get some vaccines reimbursed via the GVS but when vaccines are administered in the hospitals, they should be reimbursed via the DBC. However, the DBC reimbursements are calculated without taking vaccination in consideration, meaning that they provide insufficient reimbursement.

*“If the hospital prescribes and it is administered in the hospital, yes in that case it is a hospital activity and then there is no way to reimburse it. And yes, I think that administering by the GP is a kind of practical way to get it reimbursed for the patient”*. (Healthcare provider).

When a patient is referred to the GP from the hospital, the GP could feel he/she is forced by somebody’s hand. An emerging theme regarding affordability is that there is a lack of research about the vaccination effects in specific ICP, while the authorities require this data for the reimbursement decision. However, for the pharmaceutical industry it is not feasible to provide effectiveness data for all vaccines for every patient population.

The stakeholders recognized and agreed with the challenges indicated by the RVS and the ZIN. They indicated affordability and the knowledge deficit in healthcare providers and patients as the most important challenges. Meaning that if those challenges were solved most health gains would be achieved.

### Potential solutions

The stakeholders provided a lot of different solutions to the challenges and rarely the same solutions were mentioned. An overview of all challenges and their potential solutions in the vaccination process of ICP in the Netherlands can be found in Appendix D. Regarding a newly emerged challenge, a suggested potential solution would be to provide a fair reimbursement to stakeholders for their efforts to improve the vaccination process of ICP. Next, reimbursement of vaccines would help the inclusion of the vaccines in the guidelines according to several stakeholders. However, one authority employee indicated another potential solution to this challenge namely that healthcare providers could take more responsibility for their own healthcare quality. The translation from guidelines to practice could be improved by the creation and usage of a digital tool with an algorithm. In this tool the healthcare provider can insert the patient characteristics, the tool will then create vaccination recommendations based on the latest guidelines and data.

*“Then it is simply much more applicable in practice and GPs and healthcare providers will apply that much more.”* (Authorities).

Regarding the knowledge deficit challenge, four stakeholders mentioned that this could be solved by paying more attention to vaccinations in the education of healthcare providers. Three stakeholders indicated that clear vaccination information could be included in the guidelines for specific patient populations. According to four stakeholders, the feasibility challenge could be solved with a discussion among stakeholders about who should take the responsibility. Four stakeholders indicated that the affordability challenge could be solved by adjusting the GVS registration process, making it easier for the pharmaceutical industry to apply for reimbursement towards various high-risks groups.

The GGD employee described a clear roadmap to improve the vaccination process of ICP, starting with the reimbursement. Then the guidelines must be adjusted and aligned. Consequently, you automatically get that the focus of preventive care shifts and information is provided to patients. When the guidelines include the vaccinations, the challenge of whose responsibility it is to vaccinate is solved as well.

### Ideal scenario

**Fig. 3:**
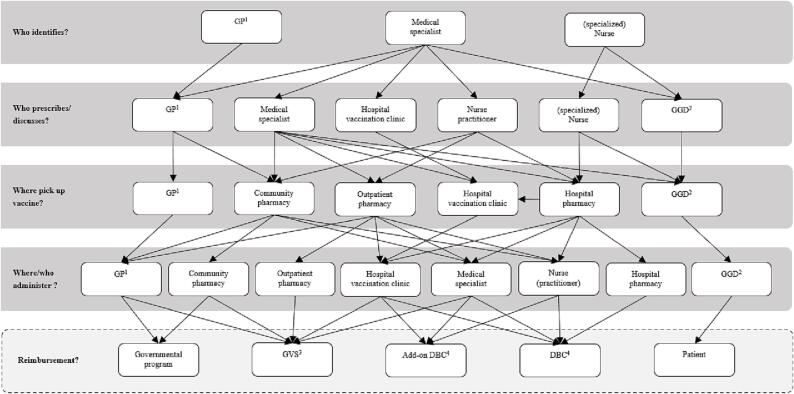
*Ideal vaccination process of ICP flowchart, according to stakeholders.*^1^GP, general practitioner; ^2^GGD, Municipal Public Health Service; ^3^GVS, medicine reimbursement system; ^4^DBC, diagnosis-treatment combination.

No clear ideal vaccination process of ICP emerged from the interviews. This is because the stakeholders have different preferences, and no preference was mentioned significantly more than another. A summary of each stakeholder’s ideal vaccination process of ICP is shown in Table 2. The variety of preferences increased with the steps of the vaccination process, which can be seen in Fig. 3. A summary of all the advantages and disadvantages of the most mentioned places to vaccinate ICP can be found in Appendix E, since this step is especially complicated.

**Table 2:** Summary of each stakeholder’s ideal vaccination process.

Stakeholder	Who identifies if a patient belongs to ICP^1^ and should receive vaccinations?	Who discusses/prescribes the vaccinations?	Where can the vaccine be picked up?	Where will the patient be vaccinated or who will vaccinate the patient?	How will the vaccine be reimbursed?
Healthcare provider	- Medical specialist	- Hospital vaccination clinic - Nurse practitioner	- Hospital	- Hospital vaccination clinic - Nurse practitioner	- GVS^2^ - Add-on DBC^3^
Healthcare provider	- Medical specialist	- Nurse practitioner	- Pharmacy	- Hospital	- DBC^3^
Healthcare provider	- Medical specialist- (specialized) Nurse	- Medical specialist- (specialized) Nurse	- Hospital pharmacy	- Hospital	- Add-on DBC^3^ - DBC^3^
Healthcare provider	- Practitioner (Medical specialist or GP^4^)	- Practitioner (Medical specialist or GP^4^)		- Hospital - GP^4^	-Reimbursement belongs to the treatment
Pharmacist	- Practitioner (Medical specialist or GP^4^)	- Practitioner (Medical specialist or GP^4^)	- Pharmacy patient usually goes to	- Treatment room of practitioner - Pharmacy patient usually goes to	- GVS^2^
Pharmaceutical industry	- Medical specialist	- Medical specialist	- Patients must pick it up themselves	- GP^4^ - Hospital vaccination clinic - Pharmacy	- GVS^2^
Pharmaceutical industry	- Medical specialist	- Medical specialist	- Hospital vaccination clinic	- Hospital vaccination clinic	- Basic health insurance
Authorities	- Practitioner (Medical specialist or GP^4^)	- Practitioner (Medical specialist or GP^4^)	-Community pharmacy - Outpatient pharmacy - Hospital pharmacy	- Community pharmacy - Outpatient pharmacy - Hospital pharmacy	- GVS^2^ - DBC^3^
Authorities	- Practitioner (Medical specialist or GP^4^)	- Practitioner (Medical specialist or GP^4^) - Nurse		- Practitioner (Medical specialist or GP^4^)	- DBC^3^
Authorities	- Practitioner (Medical specialist or GP^4^)	- Practitioner (Medical specialist or GP^4^) - Hospital vaccination clinic	- Pharmacy - GP^4^ - Hospital vaccination clinic - GGD^5^	- Practitioner (medical specialist or GP^4^) - Hospital vaccination clinic - GP^4^ - GGD^5^	- Health insurance - Out-of-pocket
Advisory body for authorities	- GP^4^	- GP^4^	- Where the patient will be vaccinated	- A qualified healthcare provider - Pharmacy	- Health insurance - Government program - Out-of-pocket
GGD^5^		- Medical specialist- (specialized) nurse - GGD^5^		- GP^4^ - GGD^5^	- Basic health insurance

1 ICP immunocompromised patients.

2 GVS medicine reimbursement system.

3 DBC diagnosis-treatment combination.

4 GP general practitioner.

5 GGD Municipal Public Health Service.

In the first step, who identifies if a patient belongs to ICP, the healthcare provider that is currently treating the patient (the practitioner) was mentioned by five stakeholders. This could be a medical specialist or a GP. Five stakeholders explicitly mentioned that the medical specialist should identify ICP. Four of those stakeholders explained that ICP are currently being treated by a medical specialist.

For the step who should discuss and/or prescribe the vaccinations most of the time the same person as who identifies the patients was mentioned. However, a nurse or a hospital vaccination clinic were mentioned as well. One healthcare provider mentioned that only the routine procedures should be done by a nurse and the more complex screenings should be done at a hospital vaccination clinic.

Two stakeholders, including the GGD employee, mentioned that the GGD could be a location to pick up and administer vaccines. The GGD employee preferred to do this at the GGD travel department, but this is currently not included in their protocols and this department cannot get reimbursements from the basic health insurance. Nevertheless, the GGD has a smooth-running vaccination process and a lot of vaccination knowledge and experience. The pharmacist mentioned that the vaccines can be picked up at the pharmacy the patient usually goes to and be administered there. Three other stakeholders mentioned this option as well. However, the current Dutch law inhibits pharmacists from administering vaccinations. Three stakeholders stated that ICP should get the vaccinations in the hospital and another four stakeholders mentioned a hospital vaccination clinic. Three healthcare providers preferred to do the whole vaccination process in the hospital. When the vaccinations are provided at the hospital this could be easily registered in an ICT-system and save an administrative burden. One healthcare provider mentioned: *“if you must outsource the administering to someone outside the hospital, you must always check whether it has been administered.”* A disadvantage of vaccinating at the hospital is that the vaccinations will be at the expense of the patient’s DBC, which is calculated without taking the vaccinations in consideration. Lastly, the ICP could be vaccinated by their GP. However, one authority employee indicated that there is already a high pressure on GPs. Despite four stakeholders think the GP is a suitable option as the GP can easier retrieve medical information of patients than other healthcare providers. Moreover, the GP practice is usually closer to the patient’s home than the hospital.

The most mentioned ways to reimburse the vaccines in the ideal vaccination process are via the GVS or via the DBC. One authority employee indicated that the place where the vaccines are picked up could determine the way of reimbursement. When the vaccines are picked up at a community or outpatient pharmacy the vaccines could be reimbursed via the GVS. And when the vaccines are picked up at the hospital pharmacy the vaccines could be reimbursed via the DBC. Two healthcare providers mentioned that it would be ideal to create a kind of add-on DBC for vaccines.

## Discussion

This study aimed to gain insights into the current challenges in the vaccination process of ICP in the Netherlands. Seven new challenges which were not stated by the RVS nor the ZIN emerged among others: stakeholders want a fair reimbursement for their efforts to improve the vaccination process; circular reasoning of vaccine reimbursement and medical guidelines; suboptimal translation from guidelines to practice; and no smooth-running infrastructure. However, most challenges corresponded with the challenges stated by the RVS and the ZIN. From the challenges indicated by the RVS, stakeholders mentioned the ICT-issues and the knowledge deficit in healthcare providers and patients the most. Feasibility, affordability, and awareness, which were indicated by the ZIN, were mentioned by the majority of the stakeholders. The stakeholders indicated affordability and the knowledge deficit in healthcare providers and patients as the most important challenges. The stakeholders rarely stated the same solutions for the challenges. A solution could be providing a fair reimbursement to stakeholders for their efforts to improve the vaccination process of ICP. Furthermore, reimbursement of vaccines would help the inclusion of vaccines in guidelines. The most important challenges could be solved by adjusting the GVS registration process and paying more attention to vaccinations in the education of healthcare providers. Furthermore, no ideal vaccination process of ICP emerged, except for the first steps. The current practitioner of the patient should identify whether the patient needs vaccinations and discuss/prescribe those.

When interpreting the results, it should be kept in mind that some challenges of the RVS and the ZIN have similarities. Furthermore, the challenge feasibility is rather broad and includes different aspects. This makes it more likely to be mentioned. Therefore this could be an alternative explanation why eleven out of the twelve participants mentioned this challenge. This study was of importance as it verified the challenges indicated by the RVS and the ZIN. Since this research was conducted with different stakeholders, broader insights into the current vaccination process of ICP have been generated and new challenges have emerged. Furthermore, the most important challenges are identified now. Other high-income countries could learn form the indicated challenges and potential solutions by considering these challenges and solutions in relation to their health policy.

The large number of challenges indicated by the RVS, the ZIN and this study implies the difficulty to solve the problem that ICP do not always receive or are not always reimbursed for specific vaccinations on which they are dependent. If ICP do not receive these vaccinations, public health benefits are missed. The number of different solutions provided for each challenge could indicated that there are different views on solving the challenges. Contrarily, this could indicate that a lot of different things need to be done. No clear ideal vaccination process could be distinguished, which shows the complexity of the problem. This research indicated the advantages and disadvantages of the most mentioned places to vaccinate ICP (see Appendix E). These findings can be complemented with literature, which are shown in Table 3.

**Table 3:** Advantages and disadvantages of most mentioned places of vaccination or persons who will vaccinate extended with literature.

Place of vaccination or person who will vaccinate	Advantages	Disadvantages
GGD^1^	- Has a lot of vaccination knowledge and experience. - At the vaccination moment everything is there, and the vaccination process runs smoothly. - During the COVID-19 pandemic the GGD^1^ showed that they are well equipped to vaccinate [40].	- Vaccinating ICP^2^ is not included in the protocols of the GGD^1^ travel department. - The GGD^1^ travel department cannot get reimbursements from the basic health insurance. - The GGD^1^ does not have access to other healthcare providers ICT^3^-systems and therefore patient information must be passed on. - Not all GGDs^1^ can be forced to provide ICP^2^ vaccinations as some GGDs^1^ might not have the workforce, knowledge or will to provide these vaccinations. - The role of the GGD^1^ in providing vaccinations to ICP^2^ is perhaps not obvious regarding the mission and tasks, and the current source of funding for this service [41]. - Providing vaccinations for ICP^2^ could lead to reimbursement problems causing that patients often need to pay it themselves [41].
Pharmacy	- In other countries pharmacist already administer vaccinations. - The pharmacy patient usually goes to has a good overview of what is going on with the patient. - Quite a few patients have a good relationship with their pharmacy. - When vaccinations are administered at the pharmacy patient usually goes to, this would save the pharmacy verification work. - Administering the vaccines at the outpatient pharmacy could be convenient for the patient as the patient is often close by the outpatient pharmacy when having a consultation/treatment in the hospital. This means no long travel time. - Both RVS^4^ and ZIN^5^ indicate that the pharmacists could play a role in providing vaccinations [11], [17]. - Pharmacy is accessible [42]. - Pharmacists could get the vaccines reimbursed via GVS^6^[28].	- The current Dutch law does not make it possible for pharmacists to administer vaccinations, but the law is currently under review. - Pharmacists have to follow trainings about administering vaccines. - Pharmacists do not know the patient enough [42].
Hospital	- Easily registered in an ICT^3^-system and saves administration burden. - Specialist has probably close contact with patient and can vaccinate during a consult. - According to ZIN^5^, the medical specialist and GP^7^ are mainly responsible for providing vaccination care for ICP^2^ in the Netherlands [28].	- The vaccinations will be at the expense of the patient’s DBC^8^, which is calculated without taking the vaccinations in consideration. - When no regular check-up, patient must go to the hospital.
Hospital vaccination clinic	- Easily registered in an ICT^3^-system and saves administration burden. - Good logistics. - Could function as a back-up consisting of experts to serve as a source of information for problem cases or complex cases.	- The vaccinations will be at the expense of the patient’s DBC^8^, which is calculated without taking the vaccinations in consideration. - When no regular check-up, patient must go to the hospital. - When vaccinations are done at the clinic this should be communicated to the practitioner and/or GP^7^.
GP^7^	- A GP^7^ can easier retrieve medical information of patients than other healthcare providers. - A GP^7^ practice is usually closer to the patient’s home than the hospital. - According to ZIN^5^, the medical specialist and GP^7^ are mainly responsible for providing vaccination care for ICP^2^ in the Netherlands [28]. - GP^7^ are qualified to administer vaccines [33]. - The GP^7^ could declare the act of administering, but the patient must purchase the vaccine [28], [30]. The vaccine could be reimbursed via the GVS^6^[28].	- Outsourcing the vaccination would lead to more of an administrative burden for the hospital. - A GP^7^ could feel he/she is forced by somebody’s hand when he/she gets a referred patient from the hospital. - It could be considered a bit strange that the GP^7^ must settle the consequences while the person who initiates the treatment claims to have nothing to do with that. - There is already a high pressure on GPs^7^. - It is now not financially appealing for GPs^7^ to provide vaccinations to ICP^2^ besides the flu vaccination. - GPs^7^ do not have clear guidelines for the vaccines that are not included in the National Immunization Program. - The National General Practitioners Association states that prevention is not the responsibility of the GP^7^[17]. So, providing vaccination is not a priority of GPs^7^[40]. - The medical specialist knowledge required for the assessment and resulting vaccination advice if often lacking among GPs^7^[28].

1 *GGD* Municipal Public Health Service.

2 *ICP* immunocompromised patients.

3 *ICT* information and communications technology.

4 *RVS* Council for Health and Society.

5 *ZIN* Dutch National Health Care Institute.

6 *GVS* medicine reimbursement system.

7 *GP* general practitioner.

8 *DBC* diagnosis-treatment combination.

In comparison, Doornekamp et al. (2019) stated that vaccinating ICP is recommended in guidelines. Contrarily, this research pointed out the circular reasoning of vaccine reimbursement and medical guidelines, which indicates that vaccines are not always included in the guidelines. Another research by Doornekamp et al. (2020) indicated that awareness of the availability of or indication for a vaccination is a cognitive determinant of ICP for vaccination uptake which is in line with the awareness and knowledge deficit challenge. Lastly, the affordability challenge is explicitly mentioned in both articles of Doornekamp et al., in the ZIN report and in this research [11], [19], [43].

A strength of this research is that all stakeholder groups in the vaccination process of ICP that could be interviewed were represented. However, only twelve interviews were conducted. It is possible that more insights could have been gained with more interviews. Another limitation is that the description of stakeholder roles could not be provided in detail due to ensuring anonymity. As the quotes of the interviewees had to be translated from Dutch to English, the confirmability of the results could have been impacted [44]. However, a member check was done to enhance this confirmability, which also increases the credibility of this research [45]. On the other hand, conducting the interviews in Dutch could be considered a strength, since the interviewees could express themselves better. Nonetheless, only one researcher analyzed the interviews, which lowers the confirmability of the findings [37]. Another researcher has checked the analysis.

The main recommendation for practice is to focus on solving the most important challenges, affordability and knowledge-deficit in healthcare providers and patients. Adjustment to the GVS registration process could make it easier for the pharmaceutical industry to apply for reimbursement towards various high-risk groups. Another recommendation is to give vaccination a more prominent role in the education of healthcare providers. This research found the challenge circular reasoning concerning vaccine reimbursement and the medical guidelines, where each stakeholder pointed to each other to solve the problem. Therefore, it is recommend that stakeholders cooperate more and discuss the problem with each other. For further research, it is recommended to examine how vaccination could be more embedded in the education of healthcare providers. Furthermore, the interviewees indicated that more research about the vaccination effects in different ICP is needed as the authorities require these data for the reimbursement decisions. This could also improve the decision-making process in other countries.

## Conclusions

This study reveals a lot of challenges in the vaccination process of ICP in the Netherlands. This implies the difficulty to solve the problem that ICP do not always receive or are not always reimbursed for specific vaccinations on which they are dependent. The challenges affordability and knowledge-deficit in healthcare providers and patients, were indicated as the most important challenges. It is recommended to focus on solving these challenges first, to achieve optimal health gains. A lot of different solutions to the challenges were mentioned indicating there is not one clear solution.. Lastly, no ideal vaccination process for Dutch ICP was found. Multiple roads lead to Rome, but each road has its own obstacles.

## Funding

This research did not receive any specific grant from funding agencies in the public, commercial, or not-for-profit sectors.

## Declaration of Competing Interest

The authors declare the following financial interests/personal relationships which may be considered as potential competing interests: C.R. de Laat was an intern at GSK when this research was conducted. J.J.M. Simons and T.A. Westra are employed by GSK. T.A. Westra holds shares in GSK. The authors declare no other financial and non-financial relationships and activities.

## Data Availability

The data that has been used is confidential.

## Appendix A Supplementary material

Supplementary data 1 Supplementary Table 1. *Interview guide*.

Supplementary data 2 Supplementary Table 2. *Predefined code list*.

Supplementary data 3 Supplementary Table 3. *Coding results*.

Supplementary data 4 Supplementary Table 4. *Challenges and potential solutions in the vaccination process of ICP*.

Supplementary data 5 Supplementary Table 5. *Advantages and disadvantages of most mentioned places of vaccination or persons who will vaccinate*.

## References

[1] Ljungman P. (2012). Vaccination of immunocompromised patients. Clin Microbiol Infect.

[2] Harpaz R., Dahl R.M., Dooling K.L. (2016). Prevalence of immunosuppression among US adults, 2013. JAMA.

[3] National Institute for Public Health and the Environment. Risicogroepen [Risk groups]; 2021. https://www.rivm.nl/ziek-door-dier/risicogroepen [accessed 4 January 2021].

[4] De Gier B., Nijsten D.R.E., Duijster J.W., Hahné S.J.M. (2017).

[5] National Health Care Institute. Advies ‘Vaccinatielandschap medische risicogroepen’ [Advice 'Vaccination landscape medical risk groups']; n.d. https://www.zorginstituutnederland.nl/werkagenda/bloed-en-immuunsysteem/advies-vaccinatielandschap-medische-risicogroepen [accessed 2 January 2021].

[6] Meidani M., Naeini A.E., Rostami M., Sherkat R., Tayeri K. (2014). Immunocompromised patients: review of the most common infections happened in 446 hospitalized patients. J Res Med Sci.

[7] Thirumala R., Ramaswamy M., Chawla S. (2010). Diagnosis and management of infectious complications in critically ill patients with cancer. Crit Care Clin.

[8] Vaudaux B.P., Bühler S., Van Delden C., Berger C., Cohen J., Powderly W.G., Opal S.M. (2017). Infectious diseases.

[9] Lopez A., Mariette X., Bachelez H., Belot A., Bonnotte B., Hachulla E. (2017). Vaccination recommendations for the adult immunosuppressed patient: a systematic review and comprehensive field synopsis. J Autoimmun.

[10] National Institute for Public Health and the Environment. Landelijke adviezen voor vaccinatie bij chronisch inflammatoire aandoeningen [National advice for vaccination in chronic inflammatory conditions]; 2020. https://lci.rivm.nl/richtlijnen/vaccinatie-bij-chronisch-inflammatoire-aandoeningen [accessed 27 January 2021].

[11] Schierenberg A., Van der Waal A., Van der Meer H. (2020).

[12] Meerveld-Eggink A., De Weerdt O., Rijkers G.T., Van Velzen-Blad H., Biesma D.H. (2008). Vaccination coverage and awareness of infectious risks in patients with an absent or dysfunctional spleen in the Netherlands. Vaccine.

[13] Bonanni P., Grazzini M., Niccolai G., Paolini D., Varone O., Bartoloni A. (2017). Recommended vaccinations for asplenic and hyposplenic adult patients. Hum Vaccin Immunother.

[14] National Institute for Public Health and the Environment. Asplenie [Asplenia]; 2018. https://lci.rivm.nl/richtlijnen/asplenie#1-inleiding [accessed 4 January 2021].

[15] Leiden University Medical Center. Stamceltransplantatie, adviezen voor thuis [Stem cell transplantation, advice for home]; 2018. https://www.lumc.nl/patientenzorg/praktisch/patientenfolders/Stamceltransplantatie-adviezen-voor-thuis [accessed 18 June 2021].

[16] Opstelten W., Bijlsma J.W., Gelinck L.B., Hielkema C.M., Verheij T.J., Van Eden W. (2016). Impaired immunity: risk groups and consequences for general practice. Ned Tijdschr Geneeskd.

[17] Council for Health and Society (2021).

[18] Blokhuis P. (2020).

[19] Doornekamp L., De Jong W., Wagener M.N., Goeijenbier M., Van Gorp E.C.M. (2019). Dutch healthcare professionals' opinion on vaccination and education to prevent infections in immunocompromised patients: a mixed-method study with recommendations for daily practice. Vaccine.

[20] Government of the Netherlands. Beleid voor veilig aanbieden van vaccinaties [Safe vaccination policy]; n.d. https://www.rijksoverheid.nl/onderwerpen/vaccinaties/beleid-voor-veilig-aanbieden-van-vaccinatie [accessed 16 January 2021].

[21] National Health Care Institute. Vaccinaties [Vaccinations]; n.d. https://www.zorginstituutnederland.nl/Verzekerde+zorg/vaccinaties [accessed 16 January 2021].

[22] National Institute for Public Health and the Environment. Griepprik [Flu shot]; 2020. https://www.rivm.nl/griep-griepprik/griepprik [accessed 17 January 2021].

[23] National Institute for Public Health and the Environment. Voor wie is de griepprik? [Who is the flu shot for?]; 2020. https://www.rivm.nl/griep-griepprik/griepprik/voor-wie-is-griepprik [accessed 17 January 2021].

[24] National Institute for Public Health and the Environment. Vaccinaties op maat [Tailor-made vaccinations]; 2020. https://www.rivm.nl/vaccinaties-op-maat [accessed 17 January 2021].

[25] Municipal Public Health Service. Waarom GGD? [Why the GGD?]; n.d. https://www.ggdreisvaccinaties.nl/waarom-ggd [accessed 19 June 2021].

[26] National Health Care Institute. Medisch-specialistische zorg (Zvw) [Medical specialist care (Healthcare law)]; n.d. https://www.zorginstituutnederland.nl/Verzekerde+zorg/medisch-specialistische-zorg-zv [accessed 16 January 2021].

[27] Tikkanen R, Osborn R, Mossialos E, Djordjevic A, Wharton GA. International health care system profiles: the Netherlands; 2020. https://www.commonwealthfund.org/international-health-policy-center/countries/netherlands [accessed 17 January 2021].

[28] National Health Care Institute. Vergoeding van extramurale geneesmiddelen (GVS) [Reimbursement of extramural medicines]; n.d. https://www.zorginstituutnederland.nl/over-ons/werkwijzen-en-procedures/adviseren-over-en-verduidelijken-van-het-basispakket-aan-zorg/beoordeling-van-geneesmiddelen/vergoeding-van-extramurale-geneesmiddelen-gvs [accessed 16 January 2021].

[29] National Health Care Institute. Beoordeling voor vergoeding van extramurale geneesmiddelen (GVS) [Assessment for reimbursement of extramural medicines]; n.d. https://www.zorginstituutnederland.nl/over-ons/werkwijzen-en-procedures/adviseren-over-en-verduidelijken-van-het-basispakket-aan-zorg/beoordeling-van-geneesmiddelen/beoordeling-extramurale-geneesmiddelen-gvs [accessed 16 January 2021].

[30] Dutch Healthcare Authority. Prestatie- en tariefbeschikking huisartsenzorg en multidisciplinaire zorg 2020 - TB/REG-20622-02 [Performance and rate decision for general practitioner care and multidisciplinary care 2020 - TB/REG-20622-02]; 2020. https://puc.overheid.nl/nza/doc/PUC_289799_22/1/ [accessed 19 June 2021].

[31] De Vries M., Kossen J. (2015).

[32] Geneesmiddelenwet [Medicines Act]. 61. The Netherlands; 2020.

[33] Wet op de beroepen in de individuele gezondheidszorg [Individual health care professions act]. 36. The Netherlands; 2020.

[34] Moser A., Korstjens I. (2018). Series: practical guidance to qualitative research. Part 3: sampling, data collection and analysis. Eur J Gen Pract.

[35] Bruins B.J. (2018).

[36] Data Protection Act. England: The Stationery Office; 1998.

[37] Shenton A.K. (2004). Strategies for ensuring trustworthiness in qualitative research projects. Educ Inf.

[38] Noble H., Mitchell G. (2016). What is grounded theory?. Evid Based Nurs.

[39] Bradley E.H., Curry L.A., Devers K.J. (2007). Qualitative data analysis for health services research: developing taxonomy, themes, and theory. Health Serv Res.

[40] Van Essen GA, Postma MJ, Wilschut JC. Corona bewijst het: Investeren in vaccinatiebeleid loont echt [Corona proves it: Investing in vaccination policy really pays off]; 2021. https://www.trouw.nl/opinie/corona-bewijst-het-investeren-in-vaccinatiebeleid-loont-echt∼b559dc02/ [accessed 16 June 2021].

[41] Institute N.H.C. (2021).

[42] Pharmaceutisch weekblad. Openbaar apothekers bieden hulp bij vaccineren [Community pharmacists offer help with vaccinations]. In: Pharmaceutisch weekblad. The Hague: Pharmaceutisch weekblad; 2021.

[43] Doornekamp L., Van Leeuwen L., Van Gorp E., Voeten H., Goeijenbier M. (2020). Determinants of vaccination uptake in risk populations: a comprehensive literature review. Vaccines (Basel).

[44] Temple B. (2008). Narrative analysis of written texts: reflexivity in cross language research. Qual Res.

[45] Yanow D., Schwartz-Shea P. (2015).

