# Supplementary material for: Vaccination process of immunocompromised patients in the Netherlands: Current challenges and potential solutions
Source: Vaccine X. 2023 Jun 27;14:100340. doi: 10.1016/j.jvacx.2023.100340 (PMC10336781; doi:10.1016/j.jvacx.2023.100340)
Supplement: Supplementary data 1 — Supplementary Table 1. Interview guide. [file mmc1.pdf]

Supplementary Table 1. *Interview guide*

**Topic**	**Interview questions**
Problem statement: *Immunocompromised patients do not always receive or are not always reimbursed for the specific vaccinations on which they are dependent.*	
Awareness	1. To what extend were you aware of this problem?
Current challenges	2. What is currently going well in the vaccination process of immunocompromised patients?
	3. Which challenges in the vaccination process of immunocompromised patients do you think there are?
	4. The Council for Health and Society (RVS) mentions in a recent report a few challenges in the vaccination process of immunocompromised patients, some of these challenges are:<br>• Currently, the focus is mainly on curative care instead of preventive care within regular healthcare.<br>• There is a knowledge deficit in healthcare providers and patients. Little attention is paid to good vaccination care. This applies to the education as well as the refresher trainings of physicians and other healthcare providers.<br>• Guidelines for GPs and medical specialist are often insufficiently up-to-date.<br>• Information to patients is frequently insufficient and inactive, especially to immunocompromised patients.<br>• There is no optimal registration of risk factors in healthcare and ICT-systems of various healthcare providers cannot communicate well with each other. This makes it difficult, for example, to target immunocompromised patients for vaccination.<br>According to a recent report of the Dutch National Health Care Institute (ZIN):<br>• Recognizability: Guidelines for vaccinations are unclear.<br>• Affordability: Some effective vaccinations are not reimbursed from the basic insurance package. Partly because there is not always data about specific immunocompromised patients available.<br>• Feasibility: There is ambiguity about which healthcare provider is responsible for providing and administering the vaccines.<br>• Awareness: Healthcare providers are not aware of the medical necessity of the vaccinations.<br><br>Do you recognize all these challenges?<br>Do you agree with these challenges?<br>According to you, which of these challenges is the most important one? So, if this challenge is solved, optimal health gains would be achieved.
	5. Are there still challenges missing?
Describing the ideal vaccination process	6. How would you describe the ideal vaccination process of the medial high-risk group?<br>So, who identifies the patient? Who prescribes the vaccinations? Where will the patient be vaccinated? Where can the vaccines be picked up? Who reimburses the vaccines?

	If time allows: Which role could the GP play in the vaccination process of immunocompromised patients?
	If time allows: Which role could the GGD play in the vaccination process of immunocompromised patients?
Potential solutions for the challenges	7. What solutions can you come up with for the challenges in the vaccination process of immunocompromised patients?
	8. Could you come up with solutions for the challenges found by the Council for Health and Society (RVS) and the Dutch National Health Care Institute (ZIN)? So, solutions to:<br>• Focus is mainly on curative care instead of preventive care within regular healthcare.<br>• There is a knowledge deficit in healthcare providers and patients. Resulting in that healthcare providers are not aware of the medical necessity of vaccinations.<br>• Guidelines for GPs and medical specialist are often insufficiently up-to-date and unclear.<br>• Information to patients is frequently insufficient and inactive, especially to immunocompromised patients.<br>• There is no optimal registration of risk factors and indication in healthcare.<br>• Some effective vaccinations are not reimbursed from the basic insurance package.<br>• There is ambiguity about which healthcare provider is responsible for providing and administering the vaccines.
	9. How could your profession contribute to further optimizing immunocompromised patients' vaccination?
Improvements for the pharmaceutical industry	10. How could the pharmaceutical industry best contribute to further optimizing immunocompromised patients' vaccination?<br>Suggestions:<br>• By conducting more (clinical) research into specific immunocompromised patients?<br>• By raising awareness of available research conducted among immunocompromised patients?<br>• By improving the medical dialogue with guidelines committees?<br>• By providing more education?<br>• By sponsoring a satellite symposium during medical conferences?

GP, general practitioner; GGD, Municipal Public Health Service; ICT, information and communications technology; RVS, Council for Health and Society; ZIN, Dutch National Health Care Institute.
