# Supplementary material for: Vaccination process of immunocompromised patients in the Netherlands: Current challenges and potential solutions
Source: Vaccine X. 2023 Jun 27;14:100340. doi: 10.1016/j.jvacx.2023.100340 (PMC10336781; doi:10.1016/j.jvacx.2023.100340)
Supplement: Supplementary data 2 — Supplementary Table 2. Predefined code list. [file mmc2.pdf]

Supplementary Table 2. *Predefined code list*

Awareness		
Going well		
Challenges stakeholder	New challenges	
	Challenges stakeholder RVS	CS Focus curative
		CS Knowledge delay
		CS Guidelines
		CS Information to patients
		CS ICT-systems
	Challenges stakeholder ZIN	CS Recognizability
		CS Affordability
		CS Feasibility
		CS Awareness
RVS and ZIN challenges	Recognizable	
	Challenges RVS	C Focus curative
		C Knowledge delay
		C Guidelines
		C Information to patients
		C ICT-systems
	Challenges ZIN	C Recognizability
		C Affordability
		C Feasibility
		C Awareness
	Most important challenge	
Ideal process	Who identifies	
	Who discusses prescribes	
	Where pick up vaccine	
	Where who vaccination	
	Reimbursement	
Solutions	Solutions new challenges	
	Solutions RVS	S Focus curative
		S Knowledge delay
		S Guidelines
		S Information to patients
		S ICT-systems
	Solutions ZIN	S Recognizability

		S Affordability
		S Feasibility
		S Awareness
Stakeholder improve	Healthcare provider	
	Pharmacist	
	Pharmaceutical industry	
	Authorities	
	Advisory body for authorities	
	GGD	
Pharma improve	Research	
	Medical dialogue	
	Education	
	Symposium	

CS, challenges stakeholder; C, challenges; GGD, Municipal Public Health Service; ICT, information and communications technology; RVS, Council for Health and Society; S, solutions; ZIN, Dutch National Health Care Institute.
