# Supplementary material for: Vaccination process of immunocompromised patients in the Netherlands: Current challenges and potential solutions
Source: Vaccine X. 2023 Jun 27;14:100340. doi: 10.1016/j.jvacx.2023.100340 (PMC10336781; doi:10.1016/j.jvacx.2023.100340)
Supplement: Supplementary data 3 — Supplementary Table 3. Coding results. [file mmc3.pdf]

**Supplementary Table 3.** *Coding results*

| **Name** | **Files** | **References** |
|---|---|---|
| Awareness | 12 | 15 |
| Period of awareness | 4 | 5 |
| Going well | 5 | 6 |
| Going well awareness | 8 | 13 |
| Going well vaccination check | 5 | 6 |
| Going well reimbursement vaccines | 5 | 5 |
| Going well national immunization program | 4 | 6 |
| Going well guidelines | 3 | 4 |
| Going well other vaccinations | 2 | 2 |
| Challenges stakeholder | 0 | 0 |
| Numerous challenges | 3 | 3 |
| New challenges | 0 | 0 |
| CS Guidelines reimbursement | 4 | 10 |
| CS Provider reimbursement | 4 | 6 |
| CS Guidelines practice | 3 | 5 |
| CS Infrastructure | 2 | 5 |
| CS Formation nurses | 1 | 7 |
| CS Cumbersome way of declaring | 1 | 2 |
| CS International collaboration | 1 | 1 |
| Challenges stakeholder RVS | 0 | 0 |
| CS ICT-systems | 6 | 14 |
| CS Knowledge deficit | 6 | 12 |
| CS Guidelines | 5 | 5 |
| CS Focus curative | 3 | 3 |
| CS Information to patients | 1 | 1 |
| Challenges stakeholder ZIN | 0 | 0 |
| CS Feasibility | 11 | 23 |
| CS Implementation of reimbursement system | 3 | 6 |
| CS Affordability | 9 | 22 |
| CS Lack of research | 3 | 6 |
| CS Awareness | 7 | 13 |
| CS Recognizability | 5 | 5 |
| RVS and ZIN challenges | 0 | 0 |
| Recognizable | 11 | 15 |

| | | |
|---|---|---|
| Challenges RVS | 0 | 0 |
| C Guidelines | 10 | 13 |
| C Focus curative | 7 | 12 |
| C ICT-systems | 6 | 15 |
| C Knowledge deficit | 6 | 10 |
| C Information to patients | 6 | 8 |
| Challenges ZIN | 0 | 0 |
| C Affordability | 10 | 20 |
| C Feasibility | 7 | 12 |
| C Awareness | 6 | 9 |
| C Recognizability | 5 | 8 |
| Most important challenge | 0 | 0 |
| MIC Knowledge deficit RVS | 6 | 7 |
| MIC Affordability ZIN | 6 | 8 |
| MIC Feasibility ZIN | 3 | 4 |
| MIC Focus curative RVS | 2 | 2 |
| MIC ICT-systems RVS | 2 | 2 |
| MIC Information to patients RVS | 2 | 2 |
| MIC Guidelines RVS | 1 | 1 |
| MIC Recognizability ZIN | 1 | 1 |
| MIC Awareness ZIN | 0 | 0 |
| Solutions | 0 | 0 |
| Solvable | 2 | 2 |
| Solutions new challenges | 0 | 0 |
| S Guideline reimbursement | 3 | 6 |
| S Guidelines practice | 3 | 3 |
| S Provider reimbursement | 3 | 3 |
| S Cumbersome way of declaring | 1 | 1 |
| S Formation nurses | 1 | 1 |
| S International collaboration | 1 | 1 |
| Solutions RVS | 0 | 0 |
| S Information to patients | 12 | 16 |
| S Knowledge deficit | 11 | 20 |
| S ICT-systems | 11 | 15 |
| S Guidelines | 10 | 18 |
| S Focus curative | 9 | 13 |
| Solutions ZIN | 0 | 0 |

| | | |
|---|---|---|
| S Feasibility | 12 | 22 |
| S Implementation of reimbursement system | 2 | 2 |
| S Affordability | 11 | 17 |
| S Lack of research | 1 | 1 |
| S Recognizability | 5 | 12 |
| S Awareness | 3 | 6 |
| Ideal process | 0 | 0 |
| Who identifies | 11 | 22 |
| Who discusses prescribes | 12 | 33 |
| Where pick up vaccine | 9 | 11 |
| Where who vaccination | 12 | 34 |
| Reimbursement | 12 | 19 |
| Ideal process GGD | 8 | 21 |
| Ideal process asked role GGD | 3 | 3 |
| Ideal process asked role GP | 3 | 3 |
| Ideal communication | 5 | 10 |
| Stakeholder improve | 0 | 0 |
| Healthcare provider | 0 | 0 |
| HP guidelines | 3 | 4 |
| HP Discuss with stakeholders | 2 | 3 |
| HP improve according to other stakeholders | 3 | 3 |
| Pharmacist | 1 | 3 |
| Pharmaceutical industry | 2 | 9 |
| Authorities | 3 | 4 |
| Authorities improve according to other stakeholders | 3 | 7 |
| Advisory body for authorities | 1 | 1 |
| GGD | 1 | 1 |
| Pharma improve | 6 | 9 |
| Research | 0 | 0 |
| Provide more research data | 8 | 8 |
| Pharma vs medical specialist research | 3 | 4 |
| Awareness research | 0 | 0 |
| Education | 0 | 0 |
| Yes education | 7 | 9 |
| No education | 2 | 3 |
| Registration | 4 | 4 |
| Availability | 3 | 4 |

| | | |
|---|---|---|
| Symposium | 3 | 4 |
| Implementation processes | 3 | 3 |
| Reasonable price | 3 | 3 |
| Pharma improve discussion stakeholders | 2 | 3 |
| Medical dialogue | 1 | 1 |
| Other | 0 | 0 |
| Nurse | 7 | 26 |
| Nurse protocol | 1 | 7 |
| Hospital vaccination clinic | 4 | 22 |
| Pharmacy vaccination | 4 | 15 |
| Follow-up vaccine | 4 | 5 |
| Tool | 3 | 6 |
| Information booklet | 3 | 6 |
| GP prevention | 2 | 4 |
| Health insurer reimbursement | 2 | 4 |
| International guidelines | 2 | 2 |

CS, challenges stakeholder; C, challenges; GGD, Municipal Public Health Service; GP, general practitioner; HP, healthcare provider; ICT, information and communications technology; MIC, most important challenge; RVS, Council for Health and Society; S, solutions; ZIN, Dutch National Health Care Institute.
