# Supplementary material for: Vaccination process of immunocompromised patients in the Netherlands: Current challenges and potential solutions
Source: Vaccine X. 2023 Jun 27;14:100340. doi: 10.1016/j.jvacx.2023.100340 (PMC10336781; doi:10.1016/j.jvacx.2023.100340)
Supplement: Supplementary data 4 — Supplementary Table 4. Challenges and potential solutions in the vaccination process of ICP. [file mmc4.pdf]

Supplementary Table 4. *Challenges and potential solutions in the vaccination process of ICP*

	Challenges (Number of stakeholders who indicated this challenge)	Potential solutions
**Newly emerged challenges**	Stakeholders want a fair reimbursement for their efforts otherwise care will always fall short of the desired quality level (4)	- Consider drawing up medical guidelines in the reimbursement systems.<br>- Fair reimbursement for HCPs who administer vaccinations.
	Circular reasoning of reimbursement of vaccines and the medical guidelines (4)	- Clarify the problem by means of discussions between the authorities and the scientific professional associations.<br>- Before registration of a product, clarify the patient indications for the vaccine in the reimbursement reports. If there is an indication, the vaccine will be included in the guidelines. If there is no indication, then this will be added as a knowledge deficit in the guidelines resulting in the conclusion that new research must be conducted.<br>- Reimburse more vaccines, which probably results in more vaccine prescriptions and faster inclusion in guidelines.<br>- Healthcare providers take more responsibility for their own healthcare quality and include vaccinations in their guidelines if they think vaccinations will be beneficial.
	Translation from guidelines to practice (3)	- The department of infectious disease control of the GGD will administer vaccinations for ICP.<br>- Protocols of National Coordination Center for Traveler Advice will be enlarged with information about ICP.<br>- Create medical guidelines that can be practically used in hospitals.<br>- Clarify the implementation of the reimbursement system.<br>- Make a patient information toolkit available for physicians.<br>- Create a digital tool with an algorithm that gives a summarized advice about the vaccinations based on inserted patient characteristics.
	No smooth-running infrastructure (2)	- Better usage of the available facilities in the hospital and creation of more awareness among hospital physicians about this.<br>- More coordination in the infrastructure.
	Lack of hospital nurses (1)	- Have sufficient hospital nurses.
	Pharmacists have a cumbersome way of declaring the vaccines (1)	- Healthcare providers include more patient information in electronic patient records and share it via the national switching point resulting in better assessment of pharmacists about a patient's right to reimbursement.
	Lack of international collaboration (1)	- Create big networks.

		- Create a platform with foreign institutes similar to the RIVM and act as an international Infectious Disease Control Center.
**Challenges indicated by the RVS**	Knowledge deficit in healthcare providers and patients (6)	- Create more awareness for vaccinations (in the professional associations).<br>- Address the knowledge deficit via professional associations.<br>- Pay more attention to vaccinations and their importance in the medicine education, in the trainings to become a healthcare provider and in the refresher trainings.<br>- Critically reflect how refresher trainings are currently organized.<br>- Include clear vaccination information in the guidelines for specific patient populations or update the guidelines.<br>- Create a hospital vaccination clinic which can provide advice to other physicians who have less knowledge about vaccinations.<br>- Healthcare providers provide information to patients during consultations.<br>- Give an information booklet about vaccinations to ICP.<br>- Provide information to patients via patient associations.<br>- Government provides more information about the importance and opportunities to protect oneself with vaccinations against other infectious diseases that are not included in the National Immunization Program.
	There is no optimal registration of risk factors in healthcare and ICT-systems of various healthcare providers cannot communicate well with each other (6)	- Extrapolate the experiences with the COVID-vaccination to other vaccinations.<br>- Create a national online vaccination registration system for adults. Transfer the vaccination data to the electronic patient records so that the data can be shared via the national switching point with other HCPs. Include an algorithm that automatically calculates when the follow-up vaccine should be administered and relate an automatic alert to this.<br>- Provide the vaccinations in the hospital so that it can be included in the electronic patient records.<br>- Healthcare providers include more patient information in the electronic patient records and share it via the national switching point with other HCPs.
	Guidelines for GPs and medical specialist are often insufficiently up-to-date (5)	- Appoint individuals who should keep the guidelines up-to-date.<br>- Collaborate internationally to keep the guidelines up-to-date.<br>- Create more focus on preventive care.<br>- Reimburse more vaccines and include these in the guidelines.

		- Medical specialists who co-wrote the RIVM guidelines could create awareness to update the guidelines of the medical specialists.<br>- Consider drawing up medical guidelines in the reimbursement systems.
	Currently, the focus is mainly on curative care instead of preventive care within regular healthcare (3)	- Include the prevention opportunities of vaccinations in the National Prevention Program.<br>- Create more awareness for prevention/vaccinations in the education and in the refresher trainings of physicians.<br>- Include prevention in guidelines.<br>- Reimburse more vaccines and include these in the guidelines.<br>- Have enough hospital nurses who could provide preventive care.<br>- Create more awareness for preventive care and make room for this in the reimbursement system.<br>- Provide preventive care in (bigger) groups and/or digitally. E.g. via Webinars.<br>- Create a bigger financial incentive to work preventively.
	Information to patients is frequently insufficient and inactive (1)	- Well-organized and clear reimbursements.<br>- Reimburse more vaccines and include these in the guidelines.<br>- Include clear vaccination information in the guidelines for specific patient populations.<br>- Discuss vaccinations during consultations.<br>- Proactively inform people about vaccinations.<br>- Have enough hospital nurses.<br>- Patient associations provide information about vaccinations to patients.<br>- Give an information booklet about vaccinations to ICP.<br>- Provide preventive care in (bigger) groups and/or digitally. E.g. via Webinars.<br>- Provide more information online via trustworthy websites.
**Challenges indicated by the ZIN**	Feasibility (11)	- Create a hospital vaccination clinic and refer patients with complex cases to the clinic before the treatment or start immunosuppressive medicine.<br>- Make an agreement between the medical specialist and GP about who takes the initiative for these vaccinations and who guards this. Report somewhere clearly whose responsibility it is to vaccinate ICP.<br>- Create awareness for prevention among GPs.<br>- Good communication between medical specialists and GPs. E.g., via a national online vaccination registration system for adults which transfers vaccination data to the electronic patient records.<br>- Indicate responsibility for vaccinating ICP in guidelines.

		- A case manager (nurse) can coordinate the preventive care for a patient under supervision of a medical specialist.<br>- The physicians who prescribes a treatment/medicine which make a person immunocompromised will take the responsibility of the consequences.<br>- Authorities could provide more direction and facilitate discussions.<br>- Discuss the problem multidisciplinary.<br>- Use hospitals where the vaccination administration is going well as an example of good practice for other hospitals.<br>- GPs or pharmacists administer the vaccine to get it reimbursed via the GVS.<br>- Medical specialists administer the vaccine. Vaccine gets reimbursed via the GVS and the administration of the vaccine gets reimbursed via the DBC of the current treatment of the patient.<br>- Vaccination is reimbursed via the GVS or via an add-on DBC or within the DBC or via government program or out-of-pocket.<br>- Have several production places of one vaccine to spread the risk of availability problems.<br>- Patients who are going to use immunosuppressive medicine will have a vaccination check with a nurse followed by vaccinations before the patient starts taking the medicine.
	Affordability (9)	- The ZIN and the VWS organize reimbursements for vaccines and prioritize this.<br>- By increasing the healthcare costs more vaccines could be reimbursed.<br>- Pharmaceutical industry applies for vaccine reimbursement for various ICP.<br>- The GVS registration process will be adjusted making it easier for the pharmaceutical industry to apply for reimbursement towards various high-risk groups.<br>- Pharmaceutical industry invests more in research about ICP.
	Awareness (7)	- Include clear vaccination information in the guidelines for specific patient populations.<br>- Pay more attention to vaccinations and their importance in the medicine education, in the trainings to become a healthcare provider and in the refresher trainings.

		- Government provides more information about the importance and opportunities to protect oneself with vaccinations against other infectious diseases that are not included in the National Immunization Program.
	Recognizability (5)	- Create medical guidelines that can be practically used in hospitals.<br>- Consider drawing up medical guidelines in the reimbursement systems.<br>- Create a hospital vaccination clinic with vaccination experts.<br>- Avoid stating in guidelines that vaccination could be considered. Just state it firmly in the guidelines.<br>- Create a digital tool with an algorithm that gives a summarized advice about the vaccinations based on inserted patient characteristics.

DBC, diagnosis-treatment combination; GP, general practitioner; GGD, Municipal Public Health Service; GVS, medicine reimbursement system; HCPs, health care professionals; ICP, immunocompromised patients; ICT, information and communications technology; RIVM, Dutch National Institute for Public Health and the Environment; RVS, Council for Health and Society; VWS, Ministry of Health Welfare and Sport; ZIN, Dutch National Health Care Institute.
