# Supplementary material for: Vaccination process of immunocompromised patients in the Netherlands: Current challenges and potential solutions
Source: Vaccine X. 2023 Jun 27;14:100340. doi: 10.1016/j.jvacx.2023.100340 (PMC10336781; doi:10.1016/j.jvacx.2023.100340)
Supplement: Supplementary data 5 — Supplementary Table 5. Advantages and disadvantages of most mentioned places of vaccination or persons who will vaccinate. [file mmc5.pdf]

Supplementary Table 5. *Advantages and disadvantages of most mentioned places of vaccination or persons who will vaccinate*

Place of vaccination or person who will vaccinate	Advantages	Disadvantages
GGD	- Has a lot of vaccination knowledge and experience.<br>- At the vaccination moment everything is there, and the vaccination process runs smoothly.	- Vaccinating ICP is not included in the protocols of the GGD travel department.<br>- The GGD travel department cannot get reimbursements from the basic health insurance.<br>- The GGD does not have access to other healthcare providers ICT-systems and therefore patient information must be passed on.<br>- Not all GGDs can be forced to provide ICP vaccinations as some GGDs might not have the workforce, knowledge or will to provide these vaccinations.
Pharmacy	- In other countries pharmacist already administer vaccinations.<br>- The pharmacy patient usually goes to has a good overview of what is going on with the patient.<br>- Quite a few patients have a good relationship with their pharmacy.<br>- When vaccinations are administered at the pharmacy patient usually goes to, this would save the pharmacy verification work.<br>- Administering the vaccines at the outpatient pharmacy could be convenient for the patient as the patient is often close by the outpatient pharmacy when having a consultation/treatment in the hospital. This means no long travel time.	- The current Dutch law does not make it possible for pharmacists to administer vaccinations, but the law is currently under review.<br>- Pharmacists have to follow trainings about administering vaccines.
Hospital	- Easily registered in an ICT-system and saves administration burden.<br>- Specialist has probably close contact with patient and can vaccinate during a consult.	- The vaccinations will be at the expense of the patient's DBC, which is calculated without taking the vaccinations in consideration.<br>- When no regular check-up, patient must go to the hospital.

Hospital vaccination clinic	- Easily registered in an ICT-system and saves administration burden.<br>- Good logistics.<br>- Could function as a back-up consisting of experts to serve as a source of information for problem cases or complex cases.	- The vaccinations will be at the expense of the patient's DBC, which is calculated without taking the vaccinations in consideration.<br>- When no regular check-up, patient must go to the hospital.<br>- When vaccinations are done at the clinic this should be communicated to the practitioner and/or GP.
GP	- A GP can easier retrieve medical information of patients than other healthcare providers.<br>- A GP practice is usually closer to the patient's home than the hospital.	- Outsourcing the vaccination would lead to more of an administrative burden for the hospital.<br>- A GP could feel he/she is forced by somebody's hand when he/she gets a referred patient from the hospital.<br>- It could be considered a bit strange that the GP must settle the consequences while the person who initiates the treatment claims to have nothing to do with that.<br>- There is already a high pressure on GPs.<br>- It is now not financially appealing for GPs to provide vaccinations to ICP besides the flu vaccination.<br>- GPs do not have clear guidelines for the vaccines that are not included in the National Immunization Program.

DBC, diagnosis-treatment combination; GP, general practitioner; GGD, Municipal Public Health Service; ICP, immunocompromised patients; ICT, information and communications technology.
